# Advances and Trends in miRNA Analysis Using DNAzyme-Based Biosensors

**DOI:** 10.3390/bios13090856

**Published:** 2023-08-29

**Authors:** Minhyuk Lee, Seungjae Kang, Sungjee Kim, Nokyoung Park

**Affiliations:** 1 Department of Chemistry, Pohang University of Science and Technology, Pohang 37673, Republic of Koreasungjee@postech.ac.kr (S.K.); 2 Department of Chemistry and the Natural Science Research Institute, Myongji University, 116 Myongji-ro, Yongin-si 17058, Republic of Korea

**Keywords:** miRNA, DNAzyme, biosensor

## Abstract

miRNAs are endogenous small, non-coding RNA molecules that function in post-transcriptional regulation of gene expression. Because miRNA plays a pivotal role in maintaining the intracellular environment, and abnormal expression has been found in many cancer diseases, detection of miRNA as a biomarker is important for early diagnosis of disease and study of miRNA function. However, because miRNA is present in extremely low concentrations in cells and many types of miRNAs with similar sequences are mixed, traditional gene detection methods are not suitable for miRNA detection. Therefore, in order to overcome this limitation, a signal amplification process is essential for high sensitivity. In particular, enzyme-free signal amplification systems such as DNAzyme systems have been developed for miRNA analysis with high specificity. DNAzymes have the advantage of being more stable in the physiological environment than enzymes, easy to chemically synthesize, and biocompatible. In this review, we summarize and introduce the methods using DNAzyme-based biosensors, especially with regard to various signal amplification methods for high sensitivity and strategies for improving detection specificity. We also discuss the current challenges and trends of these DNAzyme-based biosensors.

## 1. Introduction

miRNAs are endogenous small non-coding RNA molecules that are about 19 to 25 nucleotides in length. miRNAs play critical roles in post-transcriptional regulation of gene expression through a process called RNA interference (RNAi) [1,2]. miRNAs form complexes called RISC to inhibit the translation of mRNAs [3]. Therefore, because of their very important role in biological processes (participating in the regulation of several important biological activities), abnormal expression of miRNAs is very dangerous, and evidence has already accumulated that they are associated with several major diseases, especially cancer [4,5]. Therefore, these miRNAs are very promising as biomarkers for early diagnosis. However, since these miRNAs have short sequences and exist as miRNAs mixture with high sequence homology with very low concentrations, it is not easy to accurately detect them by traditional methods such as Northern blotting [6], RT-PCR [7], and microarrays [8]. Due to the unique characteristics of these miRNAs, miRNA analysis requires biosensing technology capable of accurate, selective, and sensitive detection.

DNAzyme is a short single-stranded DNA molecule that has the biological catalysis function [9]. DNAzymes have not been found in nature; synthetic DNAzymes have been selected using systematic evolution of ligands by exponential enrichment process (SELEX) from random sequence DNA libraries [10]. DNAzyme is widely used in biosensing platforms such as metal ion sensing and miRNA detection due to its cofactor-dependent and sequence-specific catalytic properties [11,12]. Over the past decade, DNAzyme has been in the limelight as a very powerful tool for detecting disease-related miRNAs due to several advantages: (1) DNAzyme has higher thermal and chemical stability than protein enzyme; (2) DNAzyme is easy to design to have various functions by controlling the sequence; (3) it is easy to introduce chemical synthesis and other functional groups [13]. DNAzymes mainly used for biosensing can be divided into RNA-cleaving DNAzyme (RCD) and peroxidase-mimicking DNAzyme (PMD).

### 1.1. RNA Cleaving DNAzyme (RCD)

RNA cleaving DNAzymes (RCDs) are a DNAzyme that can recognize substrate RNA and catalyze the cleavage reaction [9]. Structurally, it has an arm capable of binding substrate RNA and a circular catalytic core at both ends. RCDs can recognize and hybridize a specific substrate RNA that has a complementary sequence with the binding arm and then catalyze RNA cleavage through the catalytic core with the assistance of metal ions as a cofactor [14]. Since the sequence of the binding arm can be controlled with less effect on the catalytic function, relatively free programming and design can be performed according to the sequence of the target RNA [15]. Due to these advantages, RCDs are widely used in the biosensing field.

In particular, 8–17 and 10–23 DNAzymes are widely used in biosensing applications because they have small catalytic cores (13 nt for 8–17 DNAzyme and 15 nt for 10–23 DNAzyme) and can cleave phosphodiester bonds between unpaired purine-pyrimidine using Mg^2+^ ion as a cofactor. The cleavage properties of the two are slightly different, with the 8–17 DNAzyme being able to cleavage between N-G junctions [16] and the 10–23 DNAzyme being able to cleavage between all purine-pyrimidine junctions [17].

**Figure 1:**
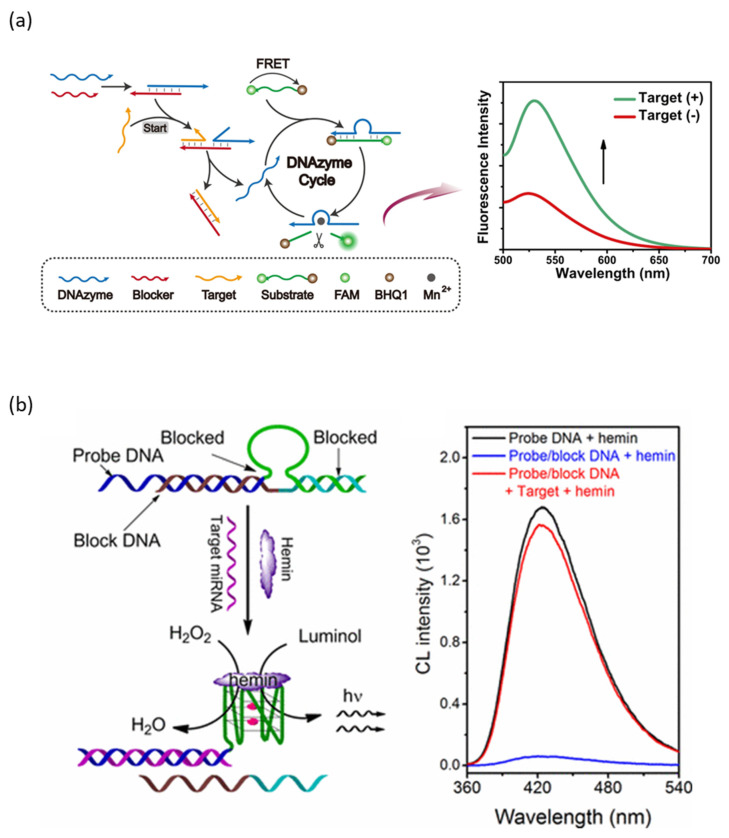
miRNA detection method using DNAzyme: (**a**) Schematic diagram of RCD-based biosensor for miRNA detection. (**b**) Schematic diagram of PMD-based biosensor for miRNA detection. (Adapted from [18,25].).

The process of detecting a target miRNA using an RCD-based probe consists of activating the catalytic core by recognizing and binding to the target miRNA, and the activated RCD catalyzes the cleavage of the reporter RNA to generate a signal (Figure 1a) [18]. RCDs normally remain inactive state and become active state only when target miRNAs are bound. When the target miRNA is bound, the catalytic core of RCD is activated by controlled binding or release through Watson-Crick base pairing. The reactivated probe can cleave the reporter RNA functionalized at both ends with an FRET pair assisted by Mn^2+^ ions, which restores the fluorescence of FAM to generate a fluorescent signal. Depending on the design of the reporter RNA cleaved by the RCD probe, not only fluorescence signals [18] but also various detection signals, such as SERS signals [19] and electrochemical signals [20], can be generated.

### 1.2. Peroxidase-Mimicking DNAzyme (PMD)

Peroxidase-mimicking DNAzymes (PMDs) are one of the most important DNAzymes for biosensing. PMD is a single-stranded DNA with a guanine-rich sequence to form a G-quadruplex structure through Hoogsteen base pairing between guanines [21]. The hemin molecule binds to this G-quadruplex as a cofactor to catalyze the oxidation reaction between H_2_O_2_ and chromogenic substrates such as ABTS [22] and PPIX [23]. Through this oxidized chromogenic substrate, signals can be analyzed through colorimetric [22], chemiluminescence [23], and electrochemical analysis [24].

Since the formation of G-quadruplexes is essential for PMD to have catalytic activity, PMD-based probes normally prevent G-quadruplexes formation for the inactivation of PMDs (Figure 1b) [25]. When the target miRNA is present, the target miRNA enables the formation of G-quadruplex through block DNA release by toehold displacement reaction, allowing reactivation of PMDs. The reactivated PMDs then bind with the hemin molecules to catalyze the oxidation of luminol to generate a chemiluminescence signal.

## 2. Signal Amplification Strategy

DNAzyme has several advantages that can be used as a sensor for various biomarkers, including miRNA, but sometimes sensitivity is not sufficient; thus, additional signal amplification strategies are needed. The strategy combining the catalytic reaction of DNAzymes and isothermal amplification reaction can obtain high amplification efficiency and has been widely used for detecting various biomarkers, including miRNA [26,27].

### 2.1. Catalytic Hairpin Assembly (CHA)

Catalytic hairpin assembly (CHA) is one of the isothermal non-enzymatic amplification techniques [28]. Generally, CHA consists of inactivated hairpins with two of each other fully complementary sequences. Opening and assembly of the two hairpins is catalyzed by the initiator, resulting in the generation of numerous double-stranded DNAs. The CHA reaction has been applied for miRNA detection due to its excellent features, including simple design, high sensitivity, and specificity. 

**Figure 2:**
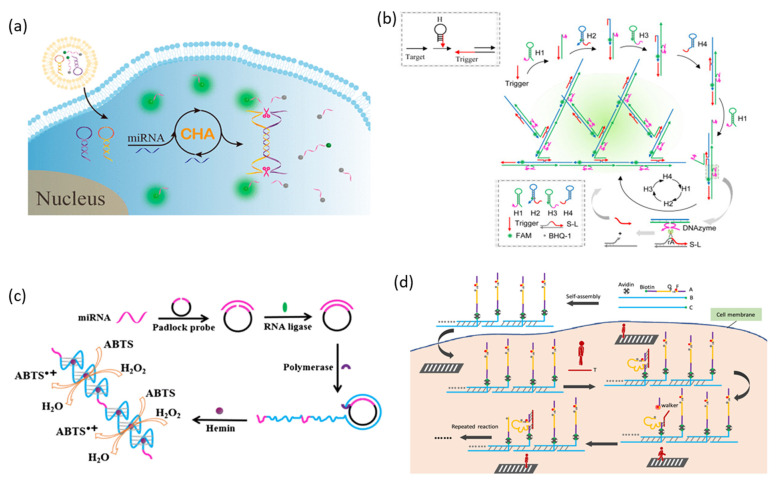
Amplification strategies based on DNAzyme for miRNA detection: (**a**) Schematic illustration of the design of the RCD-based CHA amplification method for miRNA detection. (**b**) Schematic illustration of the design of the RCD-based nonlinear HCR amplification method for miRNA detection. (**c**) Schematic illustration of the design of the PMD-based RCA amplification method for miRNA detection. (**d**) Schematic illustration of the design of the RCD-based DNA walker amplification method for miRNA detection. (Adapted from [29,32,34,37].).

The process of coupling DNAzyme and CHA for miRNA detection is that the target miRNA initiates the CHA reaction, which catalyzes the formation of DNAzyme, and DNAzyme catalyzes signal generation (Figure 2a) [29]. In this strategy, the two hairpins used to CHA were designed to partially include the catalytic core of DNAzyme. Initially, one of these hairpins is opened by the toehold displacement reaction when the target miRNA is present. Afterward, the open hairpin assembles with a second hairpin, resulting in the release of the target miRNA again, and the released target miRNA then catalyzes other hairpin assemblies to generate intact DNAzyme. The activated DNAzyme cleaves the reporter RNA functionalized with FAM and BHQ and generates a fluorescent signal.

### 2.2. Hybridization Chain Reaction (HCR)

HCR (hybridization chain reaction) is another non-enzymatic isothermal amplification technology that has a simple design and high sensitivity and selectivity [30]. HCR can be divided into linear HCR and nonlinear HCR. Linear HCR is a reaction in which, triggered by an initiator strand, a partially complementary DNA hairpin structure self-assembles into long linear double-stranded DNA. Since the chain reaction initiates only when the initiator strand presents, HCR is possible to generate an amplified signal by selective detection of the target, but the range of application is limited in traditional linear HCR due to its relatively low sensitivity. Thus, to further increase the sensitivity of HCR, nonlinear HCR has been designed. The nonlinear HCR is initiated by the initiator strand, allowing the hairpins to self-assemble into branched DNA nanostructures designed to exponentially amplify the signal [31]. Nonlinear HCR has been widely applied as a powerful signal amplification tool for biomarker detection in biosensing and bioimaging due to its simple design and operation, high sensitivity, and selectivity.

HCR-DNAzyme probe was designed to amplify the production of DNAzyme as a result of HCR in order to be applied as a biosensor for miRNA detection by coupling HCR with DNAzyme (Figure 2b) [32]. In the absence of an initiator strand, four types of hairpins coexist where the stem-loop structure is maintained stably. When the target miRNA is present, the initiator strand is opened and bound with the H1 hairpin, and the H1 hairpin structure is opened and assembled with the next hairpin continuously, triggering self-assembly, including H2, H3, and H4. Since H2 and H4 assembled here can serve as initiator strands, HCR extends into branches, resulting in the formation of a branched DNA nanostructure. Since the catalytic core of DNAzyme is divided into H1 and H3, intact DNAzymes are formed when the branched DNA nanostructure is assembled by HCR. Intact DNAzyme releases a new initiator strand that cleaves the substrate strand and induces HCR. Because the H2 hairpin has a fluorophore and quencher pair, a fluorescence signal is generated as a branched DNA nanostructure is formed.

### 2.3. Rolling Circle Amplification (RCA)

Rolling circle amplification (RCA) is an isothermal enzymatic DNA amplification process that applies rolling circle replication [33]. The RCA reaction is a reaction that uses short DNA or RNA bound to a circular DNA template as a primer to form long single-stranded DNA in which a specific DNA sequence included in the template is repeated dozens of hundreds of times in the presence of DNA polymerase. The signal amplification effect by RCA is caused by the generation of multiple repeat unit sequences. A biosensor that combines DNAzyme and RCA reaction contains a DNAzyme sequence in a circular DNA template, and DNAzyme structural units are repeatedly formed by RCA reaction. As the signal is produced by the amplified DNAzyme, the signal is amplified.

Li et al. suggested a strategy combining RCA and G-triplex PMD [34]. Here, G-triplex structures similar to G-quadruplexes can bind to hemin molecules, exhibiting the catalytic activity of PMD. However, since G-triplex requires shorter G-rich sequences, more G-triplex sequences can be included in the RCA template. Since the DNA template contains a sequence capable of recognizing the target miRNA and a G-rich sequence, the template can be hybridized by the target miRNA. Afterward, a circular DNA template can be obtained through the ligation reaction of T4 ligase, and the RCA reaction is started by phi29 DNA polymerase. The many repeating G-triplexes included in the long single-stranded DNA generated as a result of RCA combine with hemin molecules to catalyze the oxidation reaction of ABTS by H_2_O_2_ (Figure 2c).

### 2.4. DNA Walker

A DNA walker is a DNA nanomachine that moves step by step on a specific DNA track through strand displacement reaction [35]. Originally, the DNA walker was developed as a system that moves one step each time a fuel strand is inserted, but recently, it has developed into a system that walks on the DNA track autonomously and continuously. External stimuli that make DNA walkers walk autonomously are obtained from DNA strands, protein enzymes, pH changes, light, and DNAzymes. DNAzyme-powered DNA walker, called DNAzyme walker, usually takes the substrate strand as the track and DNAzyme as the walker [36]. In the general form of DNAzyme walker-based target detection, the DNAzyme is pre-inactivated and activated upon target recognition, and the DNAzyme is used as a walker to bind and cleave substrate strands step by step in the track. Substrate strand cleavage by the DNAzyme walker generates a signal. Through the coupling of DNA walker and DNAzyme cleavage reaction, it is able to obtain higher signal amplification efficiency for high sensitivity to detect miRNA existing in very small amounts.

Yang et al. used a strategy in which DNAzyme and substrate chimera strands were immobilized on a track made of DNA using biotin-streptavidin interaction, and target miRNA was used as a walker (Figure 2d) [37]. A fluorophore (Cy3) and a quencher are functionalized on either side of the cleavage site rA in the DNAzyme-substrate strand. DNAzyme activated by binding of target miRNA and DNAzyme-substrate can cleave the rA moiety of its own strand. After substrate cleavage, the distance between the fluorophore and the quencher increases, and a fluorescence signal is generated.

Table 1 shows the advantages and disadvantages of each isothermal amplification strategy introduced above.

### 2.5. Other Amplification Strategies

Furthermore, a method of amplifying a signal through a DNA- or protein-enzymatic cascade reaction using DNAzyme or protein enzyme has also been proposed.

**Figure 3:**
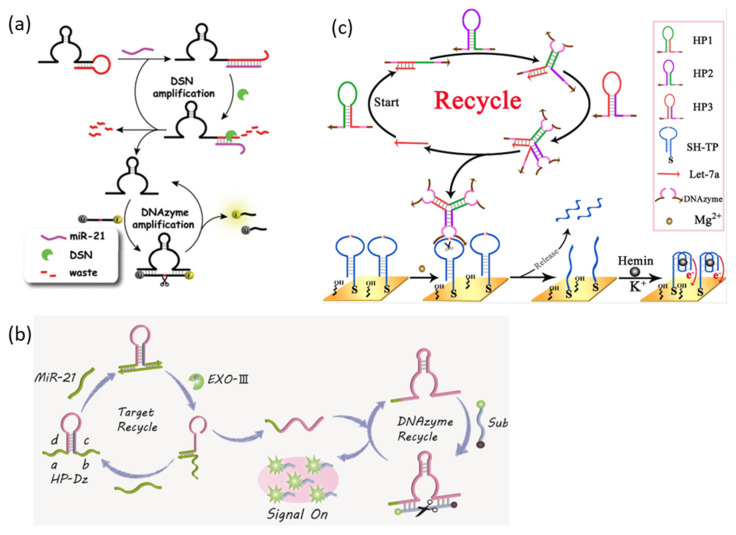
DNA- or protein-enzyme signal amplification strategy for miRNA detection. (**a**) Schematic illustration of the design of the RCD-based DSN amplification method for miRNA detection. (**b**) Schematic illustration of the design of the RCD-based Exo III assisted amplification method for miRNA detection. (**c**) Schematic illustration of the design of the dual DNAzyme-based amplification method for miRNA detection. (Adapted from [38,39,40].).

Duplex-specific nuclease signal amplification:

Duplex-specific nuclease signal amplification (DSNSA) has also introduced a method of increasing sensitivity to a target by amplifying a signal using a duplex-specific nuclease (DSN). DSN is an enzyme isolated from the hepatopancreas of Kamchatka crab. It has the characteristic of selectively cleaving only the DNA strand in the DNA-RNA hetero duplex. This is a method to catalyze the reaction that activates DNAzyme by targeting miRNA using these characteristics (Figure 3a) [38]. In this study, the DNAzyme is folded into a hairpin structure, and a DNA-RNA hetero duplex is formed by the input of the target miRNA, and the hairpin structure is opened. The DNA-RNA hetero duplex is specifically recognized by DSN, and only the DNA strand is selectively cleaved, DNAzyme is activated, and the target miRNA is released again. Released miRNAs sequentially open inactivated DNAzyme hairpins and induce activation by DSNs.

Exonuclease III-assisted signal amplification strategy:

Exonuclease III (Exo III) does not have a specific recognition site and is an enzyme that catalyzes the stepwise removal of mononucleotides from the blunt end or recessed 3′-termini of double-stranded DNA. This enzyme is not active on single-stranded DNA, and thus cleavage of 3′-protruding termini is restricted. By using these characteristics, Exo III-driven catalyzed reaction has been used as a powerful signal amplification strategy in various biosensing fields. Zhou et al. proposed a cascade reaction to amplify the signal of the DNAzyme probe to detect target miRNA using Exo III (Figure 3b) [39].

Dual DNAzyme-based miRNA sensor.

A signal amplification strategy was developed by integrating two DNAzymes, RCD and PMD, to perform roles in miRNA recognition and signal generation, respectively. For example, Ren et al. reported a signal amplification strategy in which target miRNA catalyzes the activation of RCD, RCD catalyzes PMD activation, and PMD catalyzes electrochemical signal generation (Figure 3c) [40]. The target miRNA triggers a CHA reaction that induces the formation of many trivalent RCD structures, and the activated RCDs cleave the G-rich hairpin strands on the sensor surface, resulting in the formation of a G-quadruplex that generates an electrochemical signal.

## 3. DNAzyme with Nanomaterial

The method of imparting new properties by integrating nanomaterials into DNAzyme is one of the effective strategies that are being studied extensively in the biosensor field [41]. Nanomaterials are materials with a size between 1 and 100 nm, with a large surface area and unique optical, electrical, and mechanical properties [42]. Combining the various characteristics of these nanomaterials with the catalytic characteristics of DNAzyme provides advantages such as the generation of optical signals and the role of a delivery vehicle for cell delivery [43].

### 3.1. DNA Nanostructure

Since both DNAzyme and DNA nanostructure are deoxynucleic acids, DNA nanostructure can be considered first as a delivery vehicle to increase the stability of DNAzyme. DNA nanostructure has several advantages, such as easy introduction of additional functional groups, high biocompatibility, precise structure programming, and easy formation through hybridization through annealing [44].

**Figure 4:**
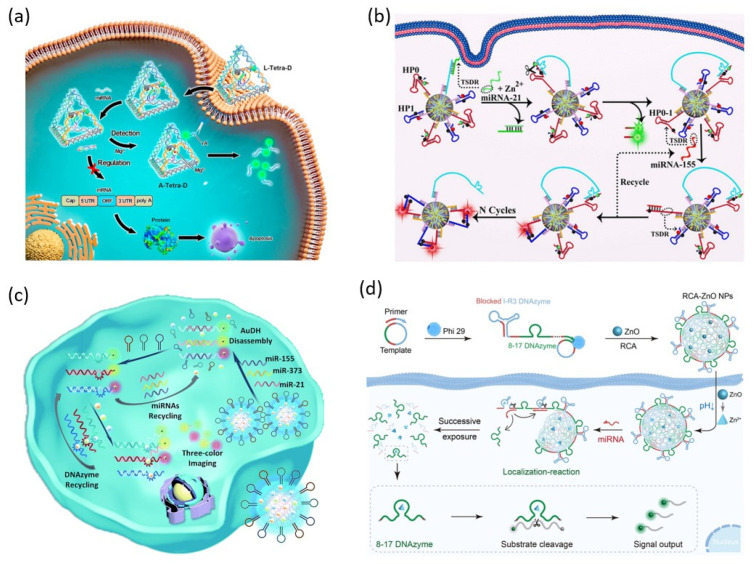
DNAzyme functionalized DNA nanostructure for miRNA detection: (**a**) Schematic illustration of oriented tetrahedron-mediated catalytic DNAzyme probe for intracellular miRNA detection. (**b**) Schematic illustration of 3D DNA nanostructure-mediated catalytic DNAzyme probe for intracellular miRNA detection. (**c**) Schematic illustration of Au-DNA nanogel-loaded catalytic DNAzyme probe for intracellular miRNA detection. (**d**) Schematic illustration of RCA-ZnO nanogel-loaded catalytic DNAzyme probe for intracellular miRNA detection. (Adapted from [46,47,49,50].).

DNA tetrahedrons have been extensively studied due to several advantages, including high biocompatibility, simple structure, high resistance to various external enzymes, and cellular uptake through receptor-mediated endocytic internalization [45]. Yu et al. proposed a DNA nanomachine containing DNAzyme in the DNA tetrahedron for miRNA detection and disease treatment in living cells (Figure 4a) [46]. The DNAzyme contained in the DNA tetrahedral nanomachines is pre-inactivated by an inhibitor strand. The inhibitor strand hybridizes with the target miRNA and is removed from the DNAzyme. The activated DNAzyme cleaves the cleavage site rA of the substrate strand coexisting in the DNA tetrahedral nanomachine in the presence of Mg^2+^ ion. The cleavage of the substrate strand increases the distance between the fluorophore and the quencher, resulting in a fluorescence signal. Simultaneously, the apoptosis of target cancer cells was induced by the target miRNA blocked by the inhibitor strand.

New 3D nanomachines synthesized in one-pot by self-assembly of several types of DNA components have attracted much attention due to their characteristics, such as low cost, rapid preparation, improved cell internalization, and high loading capacity. Li et al. recently reported the simultaneous detection of several miRNAs in living cells using 3D DNA nanomachines with large capacity (Figure 4b) [47]. This 3D DNA nanomachine achieved a shortened detection time and high sensitivity by greatly increasing the local concentration by confining the space of the loaded DNAzyme probes.

The 3D structural network DNA hydrogel has received great attention in materials science and biomedical fields due to its excellent advantages, such as high biocompatibility, programmability, biodegradability, and large loading capacity [48]. Meng et al. reported an Au-DNA hydrogel (AuDH) capable of simultaneously detecting multiple intracellular miRNAs (Figure 4c) [49]. Simultaneous detection of three miRNAs was achieved using three different DNA probes labeled with FAM, Cy3, and Cy5 fluorescent dyes and three different DNAzymes loaded in AuDH.

Recently, Shang et al. reported intracellular miRNA imaging using an RCA/ZnO nanogel probe containing two DNAzymes, a self-cleaving DNAzyme (I-R3 DNAzyme), and a signal-generating DNAzyme (8–17 DNAzyme) (Figure 4d) [50]. RCA/ZnO nanogels are used as smart nanocarriers to protect DNAzymes during intracellular delivery and enable controlled probe release by target-stimulated self-cleavage. RCA nanogel is formed by spontaneous condensation during polymerization using circular templates containing I-R3 DNAzyme and 8–17 DNAzyme. ZnO nanoparticles capable of releasing Zn^2+^ ions in a weakly acidic environment are encapsulated in RCA nanogel through electrostatic interaction to form RCA/ZnO nanogel. After the internalization of a cell, the I-R3 DNAzyme is activated by target miRNA and Zn^2+^ ion released from ZnO NPs in the acidic environment of lysosomes, and the self-cleavage process is catalyzed. Afterward, the released 8–17 DNAzyme cleaves the FAM/BHQ substrate strand, generating a fluorescence signal.

### 3.2. Metal-Organic Frameworks

Metal-organic frameworks (MOF) are solid crystalline substances in which organic ligands act as linkers between metal ions or metal clusters to form networks. MOF is a nanomaterial that is used in a wide range of fields, including photodynamic/photothermal therapy, drug delivery, and biosensing, because it is porous, has a high surface volume ratio, and is easy to functionalize [51].

**Figure 5:**
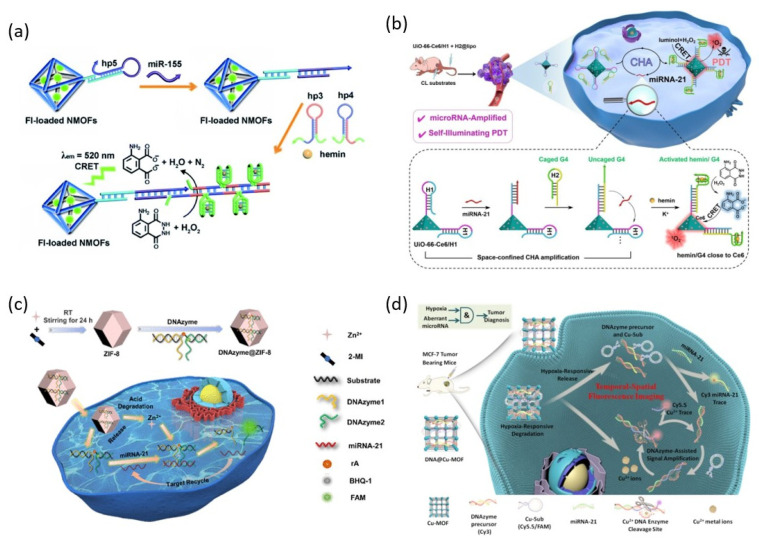
DNAzyme functionalized MOFs for miRNA detection: (**a**) Schematic illustration of catalytic PMD functionalized UiO-66 MOFs for miRNA detection. (**b**) Schematic illustration of catalytic PMD functionalized UiO-66 MOFs for intracellular miRNA detection and PDT. (**c**) Schematic illustration of catalytic RCD functionalized ZIF-8 MOFs for intracellular miRNA detection. (**d**) Schematic illustration of catalytic RCD functionalized Cu-MOF for intracellular miRNA detection. (Adapted from [52,53,54,55].).

Zhang et al. reported a dye-loaded UiO-66 MOF probe modified with PMD that detects target miRNA with a CRET-induced fluorescence signal (Figure 5a) [52]. The novelty of this system is that the fluorescence signal induced by CRET can be confined to the dye loaded on the MOF. Through this property, multiple analysis of two different miRNAs was achieved using MOFs loaded with different dyes.

Recently, a CRET-induced photodynamic therapy catalyzed by PMD in the presence of miRNAs was developed using UiO-66 MOFs loaded with photosensitizers (Figure 5b) [53]. Photodynamic therapy (PDT) is a treatment method that eliminates cancer cells with cytotoxic reactive oxygen species (ROS) produced by light irradiation to a photosensitizer. CRET-induced PDT without external laser irradiation can be an excellent solution to the problems of traditional PDT, such as insufficient tissue penetration depth of external light and photo damage to normal cells. The UiO-66 MOF loaded with the photosensitizer Ce6 is modified with an inactivated G-rich hairpin. Target miRNA triggers activation of multiple PMDs on the surface of MOF through CHA reaction. UiO-66/PMD without Ce6 loading can detect miRNA, and UiO-66-Ce6/PMD loaded with Ce6 can be used for PDT therapy.

Since the zeolite imidazole framework (ZIF-8) MOF has a positive charge on its surface, it promotes cell internalization, releases Zn^2+^ ions, and decomposes in a slightly acidic environment, making it suitable for use as a nanocarrier to deliver DNAzymes that require metal ion cofactors into live cells. Yang et al. reported the detection of miRNA in living cells using an RCD-loaded pH-responsive ZIF-8 MOF probe (Figure 5c) [54]. ZIF-8 MOF protects the RCD during delivery into cells and is degraded in a weakly acidic intracellular environment, releasing Zn^2+^ ion, a cofactor of RCDs. The pre-inactivated DNAzyme separated into two strands hybridizes with the target miRNA and catalyzes the substrate cleavage reaction that generates FAM fluorescence signals.

Since hypoxia is caused by insufficient oxygen concentration due to the rapid growth of tumors, nanomachines that recognize and respond to it have been developed to target tumors. Meng et al. achieved intracellular miRNA detection and imaging by DNAzyme-loaded Cu-MOF (DNA@Cu-MOF) that releases Cu^2+^ ions and degrades in a hypoxic tumor microenvironment (Figure 5d) [55]. In this system for hypoxic tumor cell imaging, DNA@Cu-MOF is degraded by hypoxia-induced breakage of azobenzene bridges, releasing pre-loaded Cu^2+^ ions, signal strands, and DNAzyme procurers. The signal strand is strand displaced by the target miRNA, and the Cy3 fluorescence signal is restored while the recognition site of the signal strand is exposed. This recognition site is recognized and cleaved by DNAzyme bound to the cofactor, Cu^2+^ ion. By this process, miRNA 21 is released again to start a new catalytic reaction, and the Cy 5.5 fluorescence signal is generated. 

### 3.3. Two-Dimensional Nanomaterials

#### 3.3.1. Graphene Oxide (GO)

**Figure 6:**
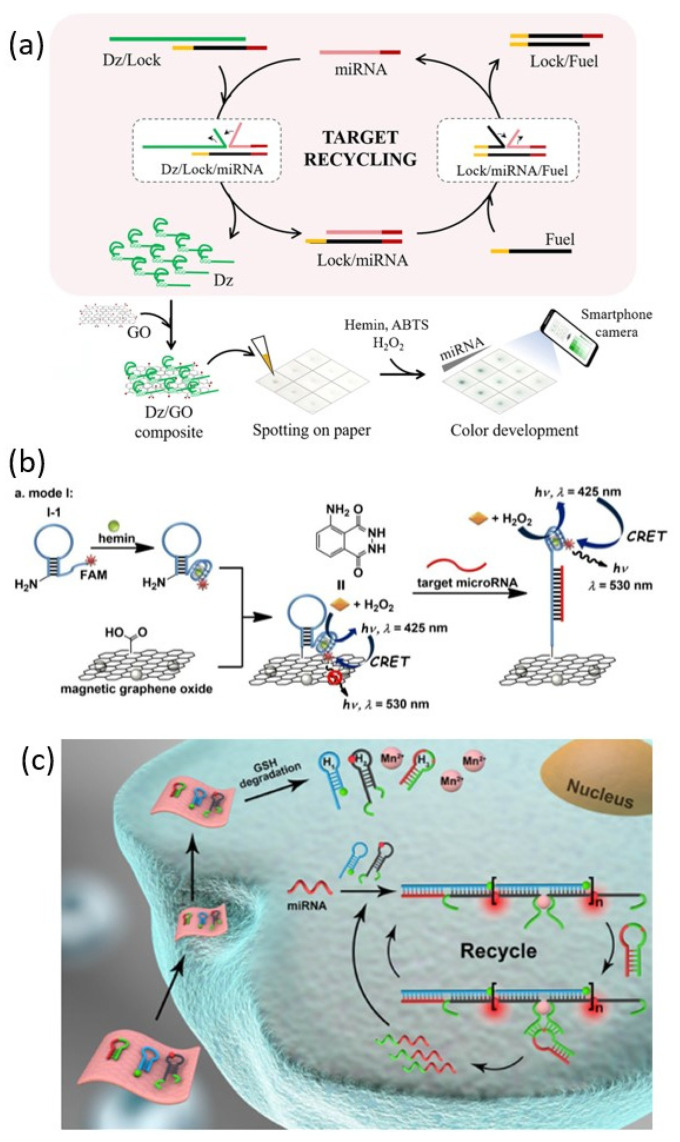
DNAzyme functionalized 2D nanomaterials for miRNA detection: (**a**) Schematic illustration of GOs-based paper sensor for miRNA detection. (**b**) Schematic illustration of catalytic PMD functionalized MGOs for miRNA detection. (**c**) Schematic illustration of catalytic DNAzyme functionalized MnO_2_ nanosheet for intracellular miRNA detection. (Adapted from [57,58,60].).

Graphene oxide (GO) is a 2D crystal of monoatomic layers of carbon obtained by oxidation of graphite. GO has an easy surface modification, high mechanical strength, water solubility, biocompatibility, and excellent electrical properties [56]. In addition, GO has the property of selectively adsorbing single-stranded DNA through π-π stacking with bases of DNA strands. Owing to these excellent properties, graphene oxide is one of the promising nanomaterials for the biomedical field. Lee et al. reported a PMD-GO composite paper-based sensor that could detect miRNA through a colorimetric method (Figure 6a) [57]. In this detection system, the target miRNA catalyzes the release of the G-rich DNAzyme (Dz) strand from the Dz/Lock duplex. The amplified Dz strand is adsorbed and collected by GO added in the next step. The Dz/GO composites are then concentrated onto paper to amplify the colorimetric response in the presence of cofactor and substrate to achieve miRNA detection.

Because GO is known as a fluorescence superquencher, it can be used as an energy acceptor for fluorescence or chemiluminescence resonance energy transfer. Bi et al. proposed a detecting system for target miRNAs through cascaded chemiluminescence resonance energy transfer (C-CRET) of a GO probe functionalized with PMD labeled with FAM using a quenching property of GO (Figure 6b) [58]. FAM is introduced for chemiluminescent energy transfer between PMD-catalyzed luminol-H_2_O_2_ and GO. Energy transfer between GO and FAM is blocked by hairpin opening through the addition of the target miRNA, and the CRET signal of FAM is generated. This process generates a miRNA detection signal. They also verified a probe that is reusable using magnetic GO and can improve sensitivity by separating the reaction and detection steps. 

#### 3.3.2. MnO_2_ Nanosheet

Two-dimensional manganese dioxide (MnO_2_) nanosheet is one of the most studied nanomaterials due to its high load capacity, biocompatibility, and degradability [59]. One of the interesting properties of the MnO_2_ nanosheets is that they react with GSH in the live cell to decompose and release a large amount of Mn^2+^ ions. Through these properties, MnO_2_ nanosheet can be used as a vehicle that can transport DNAzyme into live cells and play a role in supplying Mn^2+^ ion that can be used as a DNAzyme cofactor. Yang et al. reported miRNA imaging in living cells using MnO_2_ nanosheets loaded with three types of hairpin strands: H1, H2, and H3 (Figure 6c) [60]. MnO_2_ nanosheet acts as a carrier to deliver hairpins into living cells and then generates Mn^2+^ ion, a cofactor of DNAzyme. The linear HCR of H1 and H2 is triggered by the target miRNA, and a long double-stranded DNA structure containing DNAzymes is assembled. Activated DNAzyme catalyzes the H3 substrate strand cleavage reaction that can trigger the next HCR. The detection signal is generated by a FRET between Cy3 dye and Cy5 dye, which are located in close proximity to each other through the assembly of the long double-stranded DNA.

### 3.4. DNAzyme with Inorganic Nanoparticle

#### 3.4.1. Gold Nanoparticles

Gold nanoparticles (AuNPs) are one of the most widely used nanomaterials in many fields due to their unique optical and electronic properties, high surface-to-volume ratio, high stability, and low/non-cytotoxicity. Through thiol DNA with high affinity for the AuNP surface, it has become possible to combine the excellent properties of AuNPs with various biological functions of DNAs, and DNA-functionalized AuNPs can be applied in the biomedical field [61,62]. DNA functionalized on the surface of AuNPs is nuclease-resistant and can be easily transfected into cells. In addition, AuNPs can be used as fluorescence quenchers due to their high extinction coefficient and wide absorption range and as signal amplifiers through their surface-enhanced Raman scattering (SERS) properties.

**Figure 7:**
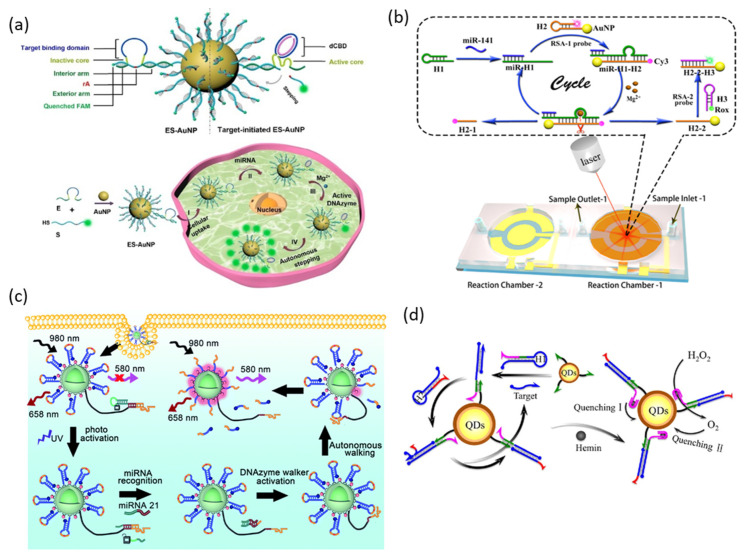
DNAzyme functionalized inorganic nanoparticles for miRNA detection: (**a**) Schematic illustration of DNAzyme functionalized AuNPs for intracellular miRNA detection. (**b**) Schematic illustration of microfluidic DNAzyme-mediated reciprocal signal amplification for miRNA detection. (**c**) Schematic illustration of DNAzyme walker functionalized UCNPs with an internal standard for precise intracellular miRNA detection. (**d**) Schematic illustration of catalytic PMD functionalized QDs for miRNA detection. (Adapted from [63,65,67,69].).

Gao et al. reported miRNA imaging in living cells using AuNPs functionalized with DNAzyme walkers and substrate strands (Figure 7a) [63]. The DNAzyme walker used in this report is pre-inactivated because the catalytic core is separated by the target binding domain. This target binding domain not only suppresses the cleavage activity of DNAzyme but also provides additional stability against DNase by providing an arch-like protective shield. Since the fluorophores functionalized on the substrate strand are quenched by AuNPs, the fluorescence signal is inhibited in the absence of miRNA. Gao et al. achieved miRNA imaging with high sensitivity in living organisms using this probe system.

The miniaturization and simplification of detection systems using microfluidic technology have been greatly studied in the past years due to their advantages, such as reduced reagent volume, high analytical throughput, and reduced space and cost [64]. Recently, Ma et al. reported accurate and rapid miRNA detection using SERS microfluidic signal amplification through DNAzyme (Figure 7b) [65]. In this study, a reciprocal signal amplification (RSA) system in which two SERS signals are reciprocally changed for reduced error and rapid analysis is suggested. This system consists of three hairpin probes: H1, H2, and H3. The H1 hairpin containing the RCD sequence is opened by recognizing the miRNA, and H2 and H3 hairpins are labeled with Cy3 and Rox, respectively. The H2 hairpin was additionally modified with a thiol group that could be functionalized on AuNP. In the normal state, the H2 strand functionalized on AuNPs forms a hairpin, and Cy3 is located in close proximity to AuNP. The H1 hairpin is opened by the target miRNA, and the DNAzyme activity is recovered and catalyzes a cleavage reaction of the H2 hairpin. The distance between Cy3 and AuNP increases, the H3 hairpin hybridizes to the AuNP surface, and Rox is located close to the AuNP. Through this series of cycle reactions triggered by the target miRNA, the two SERS signals are reciprocally changed. Further accuracy improvement and blank value reduction were achieved in the quantitative analysis of miRNAs through the absolute signal value, which is the sum of the two SERS signals.

#### 3.4.2. Upconversion Nanoparticle (UCNP)

An upconversion nanoparticle (UCNP) is a nanoparticle that exhibits anti-Stokes shift emission that can convert near-infrared (NIR) light to visible light. NIR light has great advantages for imaging living organisms because it has low photodamage and can penetrate deep into tissues due to its low scattering effect and low autofluorescence [66]. Zhang et al. reported intracellular miRNA imaging using UCNPs functionalized with DNAzyme and substrate strands (Figure 7c) [67]. The surface of UCNPs was functionalized with a DNAzyme walker that can be activated by target miRNA and a substrate strand labeled with BHQ2, and Cy3 dye was co-immobilized to efficiently transfer energy from UCNPs to BHQ2. The miRNA recognition site of DNAzyme was blocked by the photo-cleavable DNA strand, effectively suppressing false positive signals during delivery into cells. UCNPs with multiple emission properties under NIR irradiation are used as internal standards to achieve more accurate intracellular miRNA imaging.

#### 3.4.3. Semiconductor Quantum Dot

Semiconductor quantum dots (QDs) are semiconductor nanocrystals that are only a few nanometers in diameter. The most unique property of QDs is that the energy bandgap of QDs changes depending on the size of the particles, and thus the optical and electrical properties change. QDs are of great interest in bioimaging and biosensor fields due to their tunable, narrow, and strong emission; higher absorption coefficient and broad absorption range than organic phosphors; high photostability; and ease of surface modification [68]. 

Yuan et al. reported miRNA detection using PMD-functionalized QDs as probes. This biosensing system consists of two hairpins for CHA triggered by target miRNA and QDs capped with capture strands (Figure 7d) [69]. The H1 hairpin is opened by the target miRNA and hybridized with the capture strand on the QD surface, and the H2 hairpin containing the G-rich sequence replaces the target miRNA with a toehold displacement reaction. The released target miRNA catalyzes the next CHA reaction, and the hybridized H2 strand on the surface of the QDs can form a G-quadruplex and bind to hemin. QDs are doubly quenched by O_2_ generated by the catalytic activity of PMDs as well as hemin bound to G-quadruplex. Table 2 summarizes the comparison of all miRNA detection methods introduced in this review in terms of various parameters.

## 4. Conclusions and Trends

This review summarizes the various applications of DNAzyme moieties for sensitive miRNA detection and imaging to diagnose disease. DNAzym probes have various advantages, such as excellent structural stability, easy synthesis, convenient functionalization, high target specificity, and stable catalytic ability. In addition, DNAzym probes can be easily combined with various signal amplification strategies, including RCA, CHA, HCR, and DNA walkers, making it a powerful tool for disease diagnosis, biosensing, and bioimaging.

Moreover, the application of DNAzyme-based biosensors combined with various nanomaterials is also summarized. The introduction of nanomaterials has provided the following advantages: (1) Enhanced sensitivity and selectivity through response release to external stimuli such as weakly acidic pH and hypoxic environment and irradiation of UV. (2) Enhanced intracellular probe delivery efficiency through improved cellular internalization and protection of DNAzyme probes from nucleases. (3) Increased catalytic ability of DNAzyme by increasing local concentration by confining the space of DNAzyme probe. (4) Reduced reaction time and reagent volume through a simplified and miniaturized detection process. 

By utilizing the superior properties of these nanomaterials, DNAzyme-based biosensors that can simultaneously detect multiple miRNAs using multiple DNAzymes and probes have been introduced [47,49,52]. Since multiple miRNA levels are simultaneously affected in a single cancer, simultaneous detection of multiple miRNAs can increase diagnostic accuracy [70]. Theranostics, which can diagnose and treat at the same time, is a very attractive field and one of the main development directions of DNAzyme-based biosensors [71]. DNAzyme-based nanomachine theragnosis through PDT treatment or anti-miRNA treatment simultaneously with miRNA detection has been reported [46,53].

The application of DNAzyme to theragnosis has great potential, but in vivo application is not easy. The signal reporters generated by various signal amplification reactions diffuse into the cytosol, and sensitivity is reduced. In the high salt physiological environment in vivo, the activity of DNAzyme must be maintained, and the cofactor metal ion required. In addition, the shallow penetration depth of the detection signal into biological tissue is also a weakness. Although strategies have been proposed to release metal ions and DNAzyme probes after cell internalization and avoid the disadvantages of weak penetration of biological tissues through NIR light or intracellular chemiluminescence, the in vivo application of miRNA detection through DNAzyme probes is still insufficient. The potential toxicity of nanomaterials is also one of the obstacles to in vivo applications.

## Figures and Tables

**Table 1:** List of advantages and disadvantages of isothermal amplification methods.

Amplification Method	Advantages	Disadvantages
CHA	Relatively stable Simple design Low background	Less sensitivity Low reaction efficiency due to diffusion of hairpin substrate
HCR	High sensitivity Simple operation High amplification efficiency	High background due to spontaneous opening of the probes.
RCA	Generation of local amplified signals High selectivity and sensitivity	Difficulty in isolating high-purity circular templates Non-specific binding in complex water environment due to large molecular weight
DNA walker	High directionality, flexibility, sensitivity, and efficiency in vitro or in living cells	Unstable tracks within a complex biological environment. The signal amplification efficiency is greatly affected by the DNAzyme-substrate ratio.

**Table 2:** Comparison of all the miRNA detection methods introduced in this review.

Target miRNA	DNAzyme Type	Cofactor Metal Ion	Sensing Duration	Detection Limit	Read Method	Test Conditions	Ref.
miR-10b miR-21	RCD	Mn^2+^	2 h	893 pM	Fluorescence	In vitro	[18]
miR-21	PMD	K^+^	2 h	8 pM	CL	In vitro	[25]
miR-21	RCD PMD	Mg^2+^ K^+^	5 h	1 pM	Fluorescence (RCD) Colorimetry (PMD)	In cell	[29]
miR-21	RCD	Na^+^	1 h	1 pM	Fluorescence	In vitro	[32]
let-7a	PMD	K^+^	-	37 fM	Colorimetry	In vitro	[34]
miR-222	RCD	Mg^2+^	3 h	23 pM	Fluorescence	In cell	[37]
miR-21	RCD	Mg^2+^	2 h	100 fM	Fluorescence	In vitro	[38]
miR-21	RCD	Mg^2+^	4 h	100 fM	Fluorescence	In cell	[39]
let-7a	RCD PMD	Mg^2+^ K^+^	30 min	0.46 fM	Electroanalytical methods	In vitro	[40]
miR-21	RCD	Mg^2+^	2.5 h	0.77 pM	Fluorescence	In cell	[46]
miR-21 miR-155	RCD	Zn^2+^	1 h	8.2 pM (miR-21) 3.5 pM (miR-155)	Fluorescence	In cell	[47]
miR-21 miR-155 miR-373	RCD	Cu^2+^ Mg^2+^ Zn^2+^	3 h	179 aM (miR-21) 58.8 aM (miR-373) 24.9 aM (miR-155)	Fluorescence	In cell	[49]
miR-21	RCD	Zn^2+^	5 h	2 pM	Fluorescence	In cell	[50]
miR-21 miR-155	PMD	K^+^	1 h	6.7 nM (miR-21) 1.7 nM (miR-155)	CL	In vitro	[52]
miR-21	PMD	K^+^	1 h	300 pM	CL	In vivo	[53]
miR-21	RCD	Zn^2+^	4 h	47.85 pM	Fluorescence	In cell	[54]
miR-21	RCD	Cu^2+^	6 h	-	Fluorescence	In vivo	[55]
miR-122	PMD	K^+^	2 min	7.75 fmol (Solid)	Colorimetry	In vitro	[57]
miR-122	PMD	K^+^	1 h	79 pM	Fluorescence	In vitro	[58]
miR-1246	RCD	Mn^2+^	2 h	33 fM	Fluorescence	In cell	[60]
miR-21	RCD	Mg^2+^	8 h	10pM	Fluorescence	In vivo	[63]
miR-141	RCD	Mg^2+^	40 min	2.92 fM	SERS	In vitro	[65]
miR-21	RCD	Mn^2+^	2 h	3.71 pM	Fluorescence	In cell	[67]
miR-21	PMD	K^+^	1 h	37 ± 0.92 fM	Fluorescence	In vitro	[69]

## Data Availability

The data presented in this study are available upon request from the corresponding author. The data are not publicly available due to Ethical considerations.
